# Ratio of Oxygen Saturation to Inspired Oxygen, ROX Index, Modified ROX Index to Predict High Flow Cannula Success in COVID-19 Patients: Multicenter Validation Study

**DOI:** 10.5811/westjem.58311

**Published:** 2023-04-28

**Authors:** Onlak Ruangsomboon, Supawich Jirathanavichai, Nutthida Phanprasert, Chanokporn Puchongmart, Phetsinee Boonmee, Netiporn Thirawattanasoot, Thawonrat Dorongthom, Apichaya Monsomboon, Nattakarn Praphruetkit

**Affiliations:** * Mahidol University, Siriraj Hospital, Department of Emergency Medicine, Bangkok, Thailand; † Banphaeo General Hospital, Department of Emergency Medicine, Samutsakhon, Thailand; ‡ Ratchaburi Hospital, Department of Emergency Medicine, Ratchaburi, Thailand; § Buddhachinaraj Phitsanulok Hospital, Department of Emergency Medicine and Forensic Medicine, Phitsanulok, Thailand; || Prachuap Khiri Khan Hospital, Department of Emergency Medicine and Forensic Medicine, Prachuap Khiri Khan, Thailand

## Abstract

**Introduction:**

High-flow nasal cannula (HFNC) is a respiratory support measure for coronavirus 2019 (COVID-19) patients that has been increasingly used in the emergency department (ED). Although the respiratory rate oxygenation (ROX) index can predict HFNC success, its utility in emergency COVID-19 patients has not been well-established. Also, no studies have compared it to its simpler component, the oxygen saturation to fraction of inspired oxygen (SpO2/FiO2 [SF]) ratio, or its modified version incorporating heart rate. Therefore, we aimed to compare the utility of the SF ratio, the ROX index (SF ratio/respiratory rate), and the modified ROX index (ROX index/heart rate) in predicting HFNC success in emergency COVID-19 patients.

**Methods:**

We conducted this multicenter retrospective study at five EDs in Thailand between January–December 2021. Adult patients with COVID-19 treated with HFNC in the ED were included. The three study parameters were recorded at 0 and 2 hours. The primary outcome was HFNC success, defined as no requirement of mechanical ventilation at HFNC termination.

**Results:**

A total of 173 patients were recruited; 55 (31.8%) had successful treatment. The two-hour SF ratio yielded the highest discrimination capacity (AUROC 0.651, 95% CI 0.558–0.744), followed by two-hour ROX and modified ROX indices (AUROC 0.612 and 0.606, respectively). The two-hour SF ratio also had the best calibration and overall model performance. At its optimal cut-point of 128.19, it gave a balanced sensitivity (65.3%) and specificity (61.8%). The two-hour SF≥128.19 was also significantly and independently associated with HFNC failure (adjusted odds ratio 0.29, 95% CI 0.13–0.65; P=0.003).

**Conclusion:**

The SF ratio predicted HFNC success better than the ROX and modified ROX indices in ED patients with COVID-19. With its simplicity and efficiency, it may be the appropriate tool to guide management and ED disposition for COVID-19 patients receiving HFNC in the ED.

## INTRODUCTION

Coronavirus disease 2019 (COVID-19) is an infectious disease caused by the severe acute respiratory syndrome coronavirus 2 that has infected millions of individuals worldwide.^1^ Its emergence has been regarded as a worldwide public health emergency that has prompted the transformation of healthcare systems, including those of the emergency department (ED).^2^ Appropriate and effective treatment of acute hypoxemic respiratory failure (AHRF) due to COVID-19 early in the ED is essential to improve patients’ outcomes.

High-flow nasal cannula (HFNC), an oxygen-delivering technique whereby heated and humidified air is delivered with positive pressure generated, has been proven to be an effective initial respiratory support measure for patients with AHRF of many etiologies in both inpatient and ED settings.^3^–^6^ It has also been used successfully as a non-invasive airway management strategy for AHRF in COVID-19 patients.^7^,^8^ Nevertheless, HFNC therapy should always be administered with caution, as its failure may result in delayed intubation and increased mortality.^9^,^10^ Therefore, predicting HFNC success or failure and determining the optimal timing of treatment escalation to invasive mechanical ventilation are critical to avoid delayed intubation and possibly prevent mortality.

The ROX (respiratory rate oxygenation) index, a ratio of oxygen saturation (SpO_2_)/fraction of inspired oxygen (FiO_2_) to respiratory rate, has been demonstrated to be a reliable predictor of HFNC success for AHRF patients in the intensive care unit (ICU) and inpatient settings.^11^,^12^ Many studies have externally validated the ROX index as a predictor of HFNC outcomes for COVID-19 patients; however, reported results were inconsistent, possibly due to different clinical settings and cut-points employed, as well as heterogeneous population.^13^–^16^ The modified ROX index is another index incorporating heart rate (HR) to the original ROX index, which has been shown to be a good predictor of HFNC outcomes for HFNC application post-extubation.^17^ However, no studies have validated its utility in COVID-19 patients.

The SpO_2_/FiO_2_ (SF) ratio can also be employed as a predictive marker for HFNC outcomes.^18^ The SF ratio may even have superior prognostic utility to the ROX or the modified ROX indices for COVID-19 pneumonia, a specific condition in which patients usually do not present with an abnormal respiratory pattern despite severe hypoxia.^19^ Therefore, respiratory rate, a component of both types of ROX indices, may not be a good predictor of HFNC outcomes in COVID-19 patients. Although a previous study reported a superior prognosticating ability of the SF ratio over the ROX index in COVID-19 patients, it was a single-center study conducted in an inpatient setting.^20^

No studies have validated and compared the SF ratio with the ROX index or its modified version in the ED setting, where HFNC is usually initiated earlier in the disease course. Consequently, we conducted this study to evaluate and compare the prognostic utility of the SF ratio, the ROX index, and the modified ROX index in predicting HFNC success in patients with AHRF secondary to COVID-19 in the ED.

**Population Health Research Capsule**

What do we already know about this issue?

*The respiratory rate oxygenation (ROX) index is a validated predictor of high-flow nasal cannula success only in non-COVID-19 patients*.

What was the research question?

*Our goal was to compare the prognostic utility of the SpO_2_/FiO_2_ ratio, ROX, and modified ROX index in COVID-19 patients*.

What was the major finding of the study?

*The two-hour SpO_2_/FiO_2_ ratio has the best discriminative ability (AUROC 0.651, 95% CI 0.558–0.744)*.

How does this improve population health?

*For emergency COVID-19 patients, the SpO_2_/FiO_2_ ratio should be used for prognostication instead of the ROX or modified ROX indices*.

## METHODS

### Study Design and Setting

This multicenter retrospective observational study was conducted between January 1–December 31, 2021 at five EDs in Thailand. A variety of EDs from various regions of the country, including those of university hospitals and secondary- and tertiary-level hospitals, participated in the study. The five study centers were Siriraj Hospital (the nation’s largest tertiary university hospital); Banphaeo Hospital (a large general hospital); Ratchaburi Hospital (a provincial teaching hospital); Buddhachinaraj hospital (a tertiary regional advanced-level hospital); and Prachuap Khiri Khan hospital (a general standard-level hospital). The Central Research Ethics Committee of Thailand approved the study (certificate number CREC044/2022). Due to its retrospective nature, informed consent was waived. The study was reported according to the STROBE guidelines.^21^

### Participants

Adult patients over 18 years of age diagnosed with AHRF due to COVID-19 who received HFNC in the ED were included. We excluded COVID-19 patients who did not receive HFNC therapy initiated in the ED. Also excluded were those with a do-not-intubate order who received HFNC for palliative purposes.

### Study Process and Data Collection

Using International Classification of Diseases, 10^th^ Revision, codes, we retrospectively reviewed consecutive patients visiting the participating EDs with the diagnosis of COVID-19 infection made before or within the index ED visit. Their electronic health records were reviewed to determine whether they had received HFNC in the ED. We used this data if all the inclusion and exclusion criteria were satisfied. With retrospective chart review performed by trained data abstractors at each study center,^22^ we recorded the patients’ baseline characteristics, physiologic parameters, relevant blood examination results, HFNC settings, co-treatments, and important clinical outcomes.

Parameters required for calculating the SF ratio, the ROX index, and the modified ROX index were recorded before HFNC application (hour 0) and at 2 hours after HFNC initiation. All these parameters were measured while the patients were still in the ED awaiting disposition. The decisions to initiate HFNC to the patients, adjust HFNC settings, and escalate the treatment toward a more invasive respiratory support measure were determined by the attending physicians at each study center. Another study coordinator double-checked the recorded data in the electronic case-report forms to ensure the reliability and accuracy of the study data.

### Study Parameters and Outcomes

At 0 and 2 hours after HFNC initiation, we calculated three parameters and assessed them for their utility in predicting HFNC outcomes: the SF ratio; the ROX index; and the modified ROX index. The SF ratio was calculated from the ratio of SpO_2_ to FiO_2_.^18^ The ROX index was calculated from the ratio of SpO_2_/FiO_2_ to respiratory rate,^11^ and the modified ROX index was defined as the ratio of the ROX index over heart rate multiplied by 100.^17^ The primary outcome was HFNC success, defined as no requirement of mechanical ventilation following HFNC treatment at HFNC termination. The secondary outcome was overall treatment failure, defined as a requirement of escalation to mechanical ventilation following HFNC termination or mortality at hospital discharge.^23^,^24^

### Statistical Analyses

We employed descriptive statistics to describe patients’ characteristics. Categorical data is reported as frequency and percentage. Continuous variables are reported as mean and standard deviation (SD) or median and interquartile range for normally distributed and non-normally distributed data, respectively, evaluated based on histograms and Q-Q plots. We compared these variables between the success and failure groups by using the chi-squared or Fisher exact test for categorical data and an independent *t*-test or the Mann-Whitney U test for continuous data.

As an external validation study, the predictive performance of the SF ratio, the ROX index, and the modified ROX index for the study outcomes were assessed primarily with their discrimination and calibration capacities, coupled with other additional analyses.^25^ We chose the parameter with an overall superior ability over the others among all analyses performed as the best parameter in predicting the study outcomes.^25^–^27^ We reported the discrimination of each parameter with the area under the receiver operator characteristics curve (AUROC) and its 95% confidence interval (CI). We also made comparisons between the AUROCs of the study parameters for each study outcome.^28^ Calibration was reported with calibration plots and the Hosmer-Lemeshow test.^29^,^30^

Moreover, we evaluated overall model performance using the Nagelkerke R-squared. A parameter that could yield a higher R-squared value should perform better than others.^31^,^32^ We also evaluated the clinical usefulness of the parameters at the optimal cut-off values according to the Youden index by reporting their sensitivity, specificity, positive likelihood ratio, negative likelihood ratio, negative predictive value, positive predictive value, and diagnostic odds ratio. We also performed univariate and multivariate logistic regression analyses to identify independent predictors of adverse clinical outcomes: HFNC failure and overall treatment failure.

Age, gender, body mass index, day of symptoms, Charlson Comorbidity Index, Sequential Organ Failure Assessment (SOFA) score, D-dimer, C-reactive protein, and steroid were determined a priori based as existing evidence as potential associating variables with HFNC outcomes to be adjusted for in the multivariate models. We included variables with univariate *P*-value <0.2 in the multivariate regression model for each outcome. Nonetheless, each multivariate model evaluated only one potential predictor value among the three parameters at one time point to avoid multicollinearity.

We performed all statistical analyses using SPSS version 18.0 (IBM Corporation, Armonk, NY), R version 3.6.1 (R Foundation for Statistical Computing, Vienna, Austria) with the rms, Hmisc, foreign, pROC, sciplot, and dca packages, and MedCalc for Windows version 19 (MedCalc Software, Ltd, Ostend, Belgium).

## RESULTS

### Study Population

Between January 1–December 31, 2021, a total of 978 COVID-19 patients visited the participating EDs. Of these, 184 patients were treated with HFNC initiated in the ED and 11 (6%) had do-not-intubate status. Consequently, 173 patients were included in the study. Their characteristics are shown in Table 1. Of all the included patients, 92 were male (53.2%), and their mean age was 64.8±16.2 years. A total of 118 patients (68.2%) were successfully treated with HFNC, while the other 55 (31.8%) were mechanically ventilated at HFNC termination. Meanwhile, 87 patients met the criteria for overall treatment failure, and 72.4% of them had mortality at hospital discharge. The HFNC failure group had significantly higher mean age than the success group (Table 1). Otherwise, baseline demographics and initial physiologic variables were generally comparable between the two groups. Nevertheless, the failure group required higher FiO_2_ on HFNC, were on HFNC for a shorter duration, and had more complications and longer hospital length of stay than the success group (Table 1).

### Study Parameters

**Figure 1:**
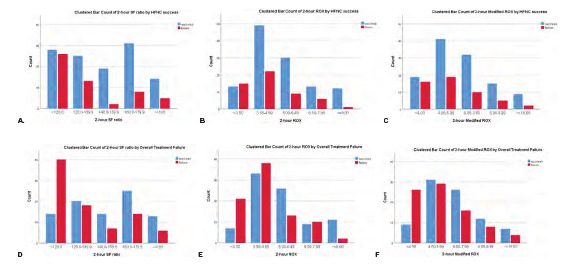
Distribution and descriptive calibration of SF ratio, ROX, and modified ROX at 2 hours for HFNC success [A, B, C] and for overall treatment failure [D, E, F]. *HFNC*, high-flow nasal cannula; *SF*, pulse oximetry/fraction of inspired oxygen ratio; *ROX*, respiratory rate oxygenation index.

There were no missing values for any parameters evaluated at either the 0- or 2-hour time points. We included all 173 samples in the analyses for both time points because no outcome events occurred prior to the two-hour time point at which the second parameter values were measured. The mean values of the three indices were lower in the HFNC failure group than in the success group; however, only the parameters measured at two hours were significantly different between the groups for both study outcomes (Table 2). Distributions of the parameter values among the study population are shown in Figure 1. For all parameters, a higher proportion of patients with higher parameter values were those with overall treatment success, implying strong associations between the parameter values and overall treatment failure (Figure 1D–1F). However, such trends and associations were not as prominent for any parameters with HFNC success (Figure 1A–1C).

### Parameters’ Performance

The ROC curves of all parameters are shown in Figure 1S, and their AUROCs are presented in Table 3. The SF ratio measured at two hours post-HFNC application had the highest discriminating capacity (AUROC 0.651, 95% CI 0.558–0.744), followed by the two-hour ROX index (AUROC 0.612, 95% CI 0.516–0.707), and two-hour modified ROX index (AUROC 0.606, 95% CI 0.512–0.700). However, none of these AUROCs were significantly different from each other (*P*-value for difference among AUROCs=0.80). Similarly, the two-hour SF ratio could yield higher AUROC for overall treatment failure than the two-hour ROX and modified ROX indices (Table 3), but these AUROCs were also not significantly different (*P*-value=0.21). The parameters measured at hour 0 all had lower discrimination than those at two hours for both study outcomes (Table 3).

**Figure 2:**
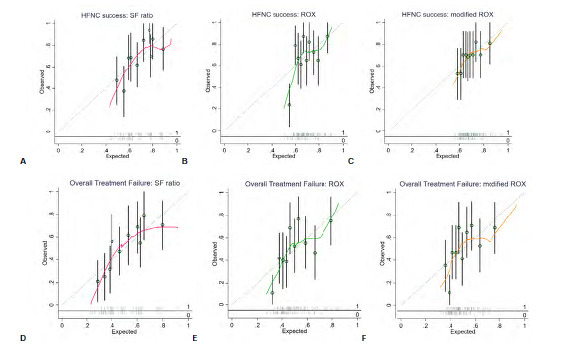
Calibration plots of SF ratio, ROX, and modified ROX at 2 hours for predicting HFNC success [A, B, C], and overall treatment failure (D, E, F]. *HFNC*, high-flow nasal cannula; *SF*, pulse oximetry/fraction of inspired oxygen ratio; *ROX*, respiratory rate oxygenation index

The two-hour SF ratio was the parameter with the best overall performance based on the Nagelkerke R-squared for both study outcomes (Table 3). Calibration based on the Hosmer-Lemeshow tests also showed that the two-hour SF ratio calibrated well with both outcomes, especially with overall treatment failure. Although the calibration plots in Figure 2 imply that there may have been an underestimation of the probability of both outcomes in the lowest and highest quintiles of probability predictions for the SF ratio that was worse than the other two parameters, these plots need to be interpreted with caution due to the small number of sample and event rates at the very low and very high predicted probabilities, possibly resulting in over/underestimation of risks due to random noise.

The two-hour SF ratio at the optimal cut-point of 128.19 for predicting HFNC success yielded the most balanced sensitivity (65.3%) and specificity (61.8%) compared to two-hour ROX≥3.23 and two-hour modified ROX≥4.27 (Table 4). Although it could also detect the lowest proportion of patients (56.6%), the rate of false positives was the lowest (21.4%) compared to the other indices. For overall treatment failure, two-hour SF ratio≥119.38 had the most balanced sensitivity and specificity and could detect the highest proportion of patients with the least false positives (Table 5).

Additionally, two-hour SF ratio was the chosen variable to be included in the multivariate regression models because it was the strongest predictor of the outcomes based on the univariate regression results (Table 5) and because it had the most superior diagnostic ability (based on Table 3 and 4) with the least input variables compared to the ROX and the modified ROX indices. From the multivariate model, two-hour SF ratio≥128.19 was the only variable independently associated with HFNC failure after adjusting for other potential confounders (adjusted odds ratio [aOR] 0.29, 95% CI 0.13–0.65; *P*=0.003) (Table 5). For overall treatment failure, the two-hour SF ratio≥119.38 was also significantly and strongly associated with the outcome (aOR 0.19, 95% CI 0.08–0.38; *P*<0.001) (Table 5).

## DISCUSSION

To the best of our knowledge, this study was the first to directly compare the prognostic utility of the SF ratio, the ROX index, and the modified ROX index for AHRF patients secondary to COVID-19 in the ED setting. We found that the SF ratio measured at two hours post-HFNC application was the best predictor of HFNC and overall treatment success since it could yield the highest discriminating ability and overall performance index, as well as good calibration, well-balanced diagnostic accuracy indices, and strong association with HFNC and overall treatment failure.

High-flow nasal cannula has been recommended as the respiratory and oxygenation support measure for patients with AHRF due to COVID-19 since it has been shown to provide many physiologic benefits and may reduce adverse outcomes, such as mechanical ventilation rate.^7^,^8^ Therefore, it has been increasingly used in many settings, including the ED, where the disease trajectory may differ from inpatient or ICU settings given that HFNC is initiated earlier in the disease course. Still, patients with failed HFNC treatment may end up requiring mechanical ventilation, and a delay in this process may result in mortality.^9^ To avoid these undesirable outcomes, it is necessary to employ adequate and appropriate patient monitoring using effective and efficient instruments.

The ROX index, the most widely validated prognostic marker of HFNC outcomes, has been shown by many studies to also have acceptable prognostic utility in COVID-19 patients.^14^–^16^, ^27^ However, only one single-center study has evaluated its utility in the ED setting.^34^ Moreover, no multicenter studies have compared the ROX index to the SF ratio, a more convenient and possibly more relevant tool for COVID-19 considering its pathophysiology and general patient characteristics, or the modified ROX index, a relatively more complex measure that also incorporates another vital sign (HR) that may be related to the disease severity and progression.

Consequently, the present study has added to the current body of evidence that the SF ratio, the simplest parameter among the three, could outperform the ROX and the modified ROX indices in predicting HFNC success for COVID-19 patients in the ED setting. Although the discrimination based on AUROC of the SF ratio was not significantly higher than those of the other parameters, it was still superior to the others by a wide range of other statistical analyses, including calibration, overall model performance, diagnostic accuracy indices based on the optimal cut-point, and associations with the outcome based on regression analyses. The superiority of the SF ratio over other more complex parameters could have been because patients with COVID-19 usually present with silent hypoxia, a condition in which other physiologic parameters can appear normal despite very low oxygenation.^19^ As a result, the study parameter with only variables relevant to oxygenation was more highly related and predictive of the outcome. The present study yielded concordant results with a study by Kim et al, who evaluated 133 COVID-19 patients receiving HFNC treatment in an inpatient setting and found that the SF ratio at one hour provided superior AUROC to that of the ROX index.^20^

However, it is important to note that although the SF ratio offered a more balanced sensitivity and specificity than the other two indices for HFNC success, the ROX index yielded a largely higher sensitivity and the proportion of detectable patients. This controversy might have been because the Youden index employed in these analyses may not be the most appropriate method to identify the optimal cut-point for this outcome as it only focused on the highest product of sensitivity combined with specificity without considering their balance. This matter was evidenced by a very high sensitivity and low specificity for the ROX index compared to the other two parameters.

Interestingly, we found that the SF ratio clearly had better predictive ability than the other indices for overall treatment failure based on all statistical analyses performed. Its performance was even better than the SF ratio for HFNC success. This result adds to the current body of evidence that mortality could have been another measure of adverse clinical outcomes of COVID-19 in patients treated with HFNC that predictive scoring systems, especially the SF ratio, could be able to predict accurately. With its unique clinical progression, disease-specific mortality could imply severe disease deterioration despite initial successful HFNC weaning.^24^

Nevertheless, despite the SF ratio having the highest AUROC, the AUROCs found in our study were generally lower than in other studies in COVID-19 patients.^14^–^16^,^20^,^33^ Such a contrast could have been explained partly by different settings and population between this study and previous studies (ED vs non-ED). Also, it could have been because of the characteristics of the population and setting specific to Thailand, a middle-income country, where the quality of care and available healthcare resources are much more limited than in other higher income countries. Our generally higher mortality and HFNC failure rates compared to other studies of higher income countries may reflect our limited-resources situation. Also, the events of in-hospital mortality and mechanical ventilation experienced by our study population might not have arisen directly due to COVID-19 but also partly due to limited healthcare provisions and suboptimal quality of care. These issues might have explained the lower discrimination capacity of the study parameters in the present study. Nevertheless, they underlie the importance of the present study as the much higher validation AUROCs in previous studies from higher income countries would not have been applicable to our scenario.

Nonetheless, from the present study, it was still appropriate to conclude that the SF ratio was superior to the ROX index and the modified ROX index in predicting HFNC success and overall treatment failure for emergency COVID-19 patients not only because of its superior performance over a wide range of statistical analytic methods but also because the SF ratio is easier to calculate at bedside, thereby being more efficient to be used in the ED.

## LIMITATIONS

There were some limitations to this study. First, the study was conducted in a middle-income country, which may limit its generalizability even though it involved multiple EDs of hospitals with varying levels of care. Second, the data was collected retrospectively, which may have caused possible errors and corresponding bias associated with the nature of a retrospective study. Third, we only measured the parameters at two hours post-HFNC application and not at any later time points because it was the longest duration that all the patients were still in the EDs; therefore, the clinical utility of the parameters could also involve aiding in ED disposition decision-making. The other reason was that there were many missing variables at later time points because the physiologic parameters were not monitored simultaneously among inpatient units of participating hospitals. Nevertheless, had the parameters been followed for longer than two hours, their prognostic utility and their relative ability in prognostication could have changed. Regardless, the applicability of those findings to the ED setting may be limited.

## CONCLUSION

The SF ratio measured two hours after high-flow nasal cannula initiation was better than the ROX index and the modified ROX index at predicting HFNC success in patients with acute hypoxemic respiratory failure secondary to COVID-19 in the ED setting. Compared to the other two ROX indices, the two-hour SF ratio had the greatest prognostic utility, as well as the utmost simplicity and bedside efficiency. Therefore, it may be an appropriate tool to guide appropriate disposition, further management, and potential escalation therapy for COVID-19 patients treated with HFNC therapy in the ED.

## Supplementary Information



## Figures and Tables

**Table 1:** Patient characteristics by high-flow nasal cannula success status.

Characteristic	Success (n=118)	Failure (n=55)	*P*-value
Gender, male	61 (51.7)	31 (56.4)	0.57
Age, years	66.3±17.1	61.2±13.6	0.04
Body mass index (kg/m^2^)	27.3±7.1	27.5±7.7	0.85
Day of symptoms upon arrival	5.5 [4.3]	5 [4.0]	0.50
Underlying diseases			
Chronic pulmonary disease	13 (11.0)	5 (9.1)	0.70
Cardiovascular disease	17 (14.4)	8 (14.5)	0.98
Diabetes mellitus	45 (38.1)	19 (34.5)	0.65
Chronic kidney disease	16 (13.6)	12 (21.8)	0.17
Charlson Comorbidity Index	1 [3]	1 [3]	0.40
Initial vital signs			
Systolic blood pressure	140.8±27.4	144.0±30.9	0.50
Diastolic blood pressure	79.0±15.9	81.7±17.6	0.31
Pulse rate	94.6±20.1	98.0±21.5	0.31
Respiratory rate	35.2±8.1	35.5±6.3	0.79
Pulse oximetry	86 [13.5]	82.5 [15.0]	0.09
Glasgow Coma Scale	15 [0]	15 [0]	0.21
Initial blood examination			
White blood cells (x1000/mm^3^)	7.8 [4.6]	8.9 [4.8]	0.52
Platelet (x1,000/mm^3^)	240.1±113.6	226.1±98.2	0.44
Glomerular filtration rate (mL/min)	68.8±33.2	65.3±30.6	0.51
D-dimer (mg/L)	1.2 [2.6]	1.1 [1.8]	0.22
C-reactive protein (mg/L)	108.1 [99.5]	106.3 [92.8]	0.58
Sequential Organ Failure Assessment score	3.0±1.9	2.9±1.6	0.71
HFNC settings			
Temperature (ºC)	34 [3]	34 [3]	0.79
Flow (L/min)	51.2±6.0	51.6±4.8	0.67
Fraction of inspired oxygen	0.67±0.13	0.75±0.15	0.001
HFNC treatment duration, day	6.2 [5.5]	1.9 [3.9]	<0.001
Co-treatment			
Steroid	115 (97.5)	55 (100)	0.23
Favipiravir	113 (95.8)	52 (94.5)	0.72
Remdesivir	41 (34.7)	27 (49.1)	0.07
Tocilizumab	16 (13.6)	9 (16.4)	0.63
Vasopressor	10 (8.5)	34 (61.8)	<0.001
Continuous renal replacement therapy	7 (5.9)	8 (14.5)	0.06
Complication			
Bacterial pneumonia	32 (27.1)	22 (40.0)	0.09
Acute respiratory distress syndrome	14 (11.9)	41 (74.5)	<0.001
Septic shock	12 (10.2)	33 (60.0)	<0.001
ICU admission	25 (21.2)	44 (80.0)	<0.001
ED length of stay, hour	24 [40]	26 [40]	0.80
Hospital length of stay, day	11 [9]	17 [18]	<0.001
Hospital mortality	32 (27.1)	31 (56.4)	<0.001

Note: Data is presented as frequency (percentage), mean ± SD or median [interquartile range] as appropriate.

*HFNC*, high-flow nasal cannula; *ICU*, intensive care unit; *ED*, emergency department.

**Table 2:** Descriptive statistics of potential predictors of high-flow nasal cannula outcomes.

Parameter	Time-point (hours)	HFNC success			Overall treatment failure		
		Success (n=118)	Failure (n=55)	P-value	Success (n=86)	Failure (n=87)	P-value
SpO_2_/FiO_2_ ratio	0	193.44±66.90	180.44±70.63	0.25	197.04±65.54	187.71±70.22	0.14
	2	147.57±31.17	130.28±30.39	0.001	151.96±30.46	132.14±30.28	<0.001
ROX	0	5.92±2.56	5.26±2.13	0.08	6.0±2.43	5.42±2.43	0.12
	2	5.27±1.81	4.53±1.52	0.01	5.50±1.87	4.57±1.49	<0.001
Modified ROX	0	6.91±3.49	5.90±2.83	0.06	6.97±3.45	6.20±3.15	0.13
	2	6.38±2.86	5.42±2.21	0.03	6.72±2.97	5.44±2.25	0.002

*HFNC*, high-flow nasal cannula; *SpO_2_*, pulse oximetry; *FiO_2_*, fraction of inspired oxygen; *ROX*, respiratory rate oxygenation index.

**Table 3:** Prognostic performance of the parameters before and after high-flow nasal cannula (HFNC) application in predicting HFNC success and overall treatment failure.

Parameter	Before HFNC (0 hour)				2 hours after HFNC			
	Overall performance	Calibration	Discrimination		Overall performance	Calibration	Discrimination	
	Nagelkerke R-Square (%)	Hosmer-Lemeshow test	AUROC (95%CI)	p-value	Nagelkerke R-square (%)	Hosmer-Lemeshow test	AUROC (95%CI)	P-value
HFNC success								
SpO_2_/FiO2 ratio	1.2	0.021	0.603 (0.510–0.695)	0.029	9.4	0.321	0.651 (0.558–0.744)	0.001
ROX	2.4	0.730	0.586 (0.495–0.677)	0.063	5.8	0.005	0.612 (0.516–0.707)	0.022
Modified ROX	3.1	0.216	0.593 (0.500–0.686)	0.049	4.2	0.939	0.606 (0.512–0.700)	0.026
Overall treatment failure								
SpO_2_/FiO_2_ ratio	1.7	0.086	0.616 (0.529–0.703)	0.009	13.0	0.604	0.692 (0.612–0.771)	<0.001
ROX	1.9	0.221	0.592 (0.504–0.679)	0.039	9.6	0.030	0.649 (0.565–0.732)	<0.001
Modified ROX	1.8	0.213	0.569 (0.481–0.656)	0.121	7.9	0.087	0.647 (0.563–0.731)	0.001

Note: *P*-value for differences in AUROC among any parameters for HFNC success = 0.799, for overall treatment failure = 0.213.

*HFNC*, high-flow nasal cannula; *SpO2*, pulse oximetry; *FiO2*, fraction of inspired oxygen; *ROX*, respiratory rate oxygenation index.

**Table 4:** Diagnostic accuracy indices of the parameters measured two hours after application of high-flow nasal cannula.

Parameter	N (%) [false positive (%)]	Sensitivity (%)	Specificity (%)	PPV (%)	NPV (%)	LR+	LR−	DOR
HFNC success								
SF ratio≥128.19	98 (56.6) [21 (21.4)]	65.3 (55.9, 73.8)	61.8 (47.7, 74.6)	78.6 (69.1, 86.2)	45.3 (33.8, 57.3)	1.7 (1.2, 2.5)	0.6 (0.4, 0.8)	3.0 (1.6, 5.9)
ROX≥3.23	157 (90.8) [42 (26.8)]	97.5 (92.7, 99.5)	23.6 (13.2, 37.0)	73.2 (65.6, 80.0)	81.3 (54.4, 96.0)	1.3 (1.1, 1.5)	0.1 (0.03, 0.4)	11.9 (3.4, 40.6)
Modified ROX≥4.27	127 (73.4) [34 (26.8)]	78.8 (70.3, 85.8)	38.2 (25.4, 52.3)	73.2 (64.6, 80.7)	45.7 (30.9, 61.0)	1.3 (1.0, 1.6)	0.6 (0.3, 0.9)	2.3 (1.2, 4.6)
Overall treatment failure								
SF ratio≥119.38	126 (72.8) [50 (39.7)]	88.4 (79.7, 94.3)	42.5 (32.0, 53.6)	60.3 (51.2, 68.9)	78.7 (64.3, 89.3)	1.5 (1.3, 1.9)	0.3 (0.2, 0.5)	5.6 (2.6, 12.2)
ROX≥4.36	103 (59.5) [40 (38.8)]	73.3 (62.6, 82.2)	54.0 (43.0, 64.8)	61.2 (51.1, 70.6)	67.1 (54.9, 77.9)	1.6 (1.2, 2.1)	0.5 (0.3, 0.7)	3.2 (1.7, 6.1)
Modified ROX≥4.06	107 (61.8) [43 (40.2)]	74.4 (63.9, 83.2)	50.6 (39.6, 61.5)	59.8 (49.9, 69.2)	66.7 (54.0, 77.8)	1.5 (1.2, 1.9)	0.5 (0.3, 0.8)	3.0 (1.6, 5.6)

*HFNC*, high-flow nasal cannula; *SF*, ratio of oxygen saturation (SpO2)/fraction of inspired oxygen (FiO2); *ROX*, respiratory rate oxygenation index; *PPV*, positive predictive value; *NPV*, negative predictive value; *LR*, likelihood ratio; *DOR*, diagnostic odds ratio.

**Table 5:** Univariate and multivariate analyses of factors predicting high-flow nasal cannula outcomes.

Variable	HFNC failure		Overall treatment failure	
	Univariate OR (95% CI; P-value)	Multivariate OR (95% CI; P-value)	Univariate OR (95% CI; P-value)	Multivariate OR (95% CI; P-value)
Age (per 1-year increase)	0.98 (0.96–1.04; P=0.25)	-	1.03 (1.01–1.05; P=0.005)	1.02 (0.99–1.04; P=0.23)
Gender male (vs female)	1.21 (0.63–2.30; P=0.57)	-	1.18 (0.65–2.14; P=0.60)	-
Body mass index (kg/m^2^)	1.0 (0.96–1.05; P=0.85)	-	0.97 (0.93–1.01; P=0.11)	0.98 (0.93–1.04; P=0.50)
Day of symptoms on arrival	0.95 (0.86–1.05; P=0.31)	-	0.91 (0.83–0.99; P=0.04)	0.92 (0.82–1.03; P=0.16)
Charlson Comorbidity Index	1.04 (0.91–1.18; P=0.59)	-	1.12 (0.98–1.27; P=0.09)	1.03 (0.87–1.21; P=0.74)
Sequential Organ Failure Assessment score (per 1-point increase)	0.96 (0.79–1.17; P=0.71)	-	1.34 (1.10–1.64; P=0.004)	1.19 (0.95–1.49; P=0.14)
D-dimer≥1.5mg/L (vs <1.5 mg/L)	0.57 (0.26–1.26; P=0.17)	0.52 (0.23–1.19; P=0.12)	1.13 (0.55–2.33; P=0.74)	-
C-reactive protein (vs <100 mg/L)	Ref	-	Ref	-
100–200 mg/L	0.94 (0.45–1.94; P=0.87)		1.32 (0.67–2.58; P=0.43)	
>200 mg/L	1.20 (0.46–3.09; P=0.71)		1.38 (0.56–3.43; P=0.49)	
Steroid	n/a (too few observations)	-	2.05 (0.18–23.01; P=0.56)	-
0-hour SpO_2_/FiO_2_ ratio	1.0 (0.99–1.0; P=0.25)	-	1.0 (0.99–1.0; P=0.14)	-
0-hour ROX	0.88 (0.76–1.03; P=0.10)	-	0.91 (0.80–1.03; P=0.12)	-
0-hour modified ROX	0.90 (0.80–1.01; P=0.07)	-	0.93 (0.85–1.02; P=0.14)	-
2-hour SpO_2_/FiO_2_ ratio ≥ optimal cut-point (vs < optimal cut-point)^a^	0.32 (0.16–0.62; P=0.001)	0.29 (0.13–0.65; P=0.003)	0.17 (0.08–0.38; P<0.001)	0.19 (0.08–0.46; P<0.001)
2-hour ROX ≥ optimal cut-point (vs < optimal cut-point)^b^	0.38 (0.17–0.71; P=0.002)	-	0.29 (0.15–0.56; P<0.001)	-
2-hour modified ROX; ≥ optimal cut-point (vs < optimal cut-point)^c^	0.41 (0.20–0.82; P=0.01)	-	0.25 (0.11–0.56; P=0.001)	-

Note:

a Optimal cut-points of SpO2/FiO2 ratio were 128.19 for HFNC failure and 119.38 for overall treatment failure,

b optimal cut-points of ROX were 3.23 for HFNC failure and 4.36 for overall treatment failure,

c optimal cut-points of Modified ROX were 4.27 for HFNC failure and 4.06 for overall treatment failure.

Variables with univariate *P*-value< 0.2 were included in the multivariate logistic regression models. Only one strongest parameters in predicting each outcome among SpO2/FiO2 ratio, ROX, and modified ROX were included in the multivariate models to avoid multicollinearity.

*OR*, odds ratio; *CI*, confidence interval; G*FR*, glomerular filtration rate; *SpO2*, pulse oximetry; *FiO2*, fraction of inspired oxygen; *ROX*, respiratory rate oxygenation index.
