# Supplementary material for: Ratio of Oxygen Saturation to Inspired Oxygen, ROX Index, Modified ROX Index to Predict High Flow Cannula Success in COVID-19 Patients: Multicenter Validation Study
Source: West J Emerg Med. 2023 Apr 28;24(3):511–21. doi: 10.5811/westjem.58311 (PMC10284520; doi:10.5811/westjem.58311)
Supplement: Supplementary file 1 [file wjem-24-511-s001.docx]

**Figure 1S. Receiver operating characteristics curves (ROC) of potential predictors of high-flow nasal cannula outcomes**

A
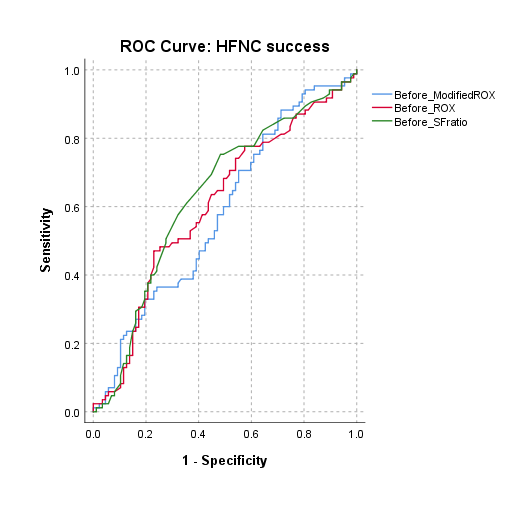
B
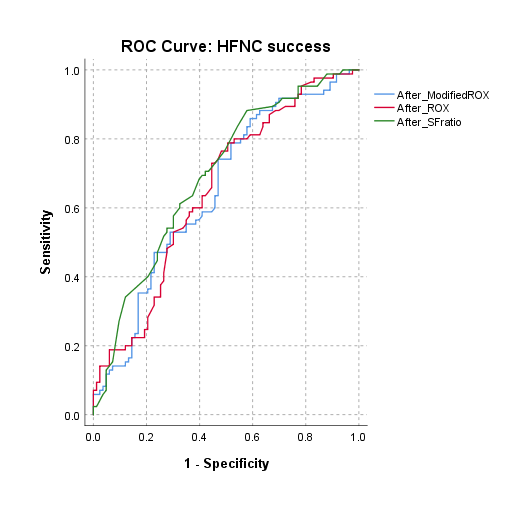


C
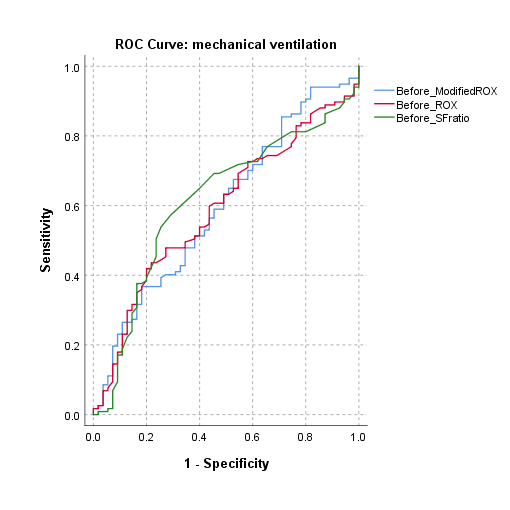
 D
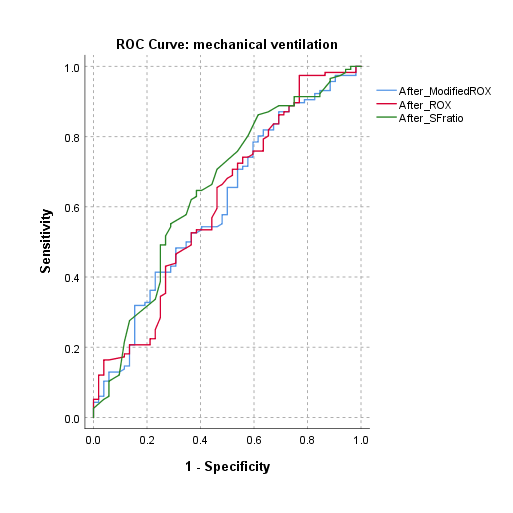


Note: (A) ROC curves of predictors measured before HFNC application for HFNC success, (B) ROC curves of predictors measured 1-2 hours after HFNC application for HFNC success,

(C) ROC curves of predictors measured before HFNC application for no mechanical ventilation, (D) ROC curves of predictors measured 1-2 hours after HFNC application for no mechanical ventilation

Abbreviations: HFNC, high-flow nasal cannula; SF; pulse oximetry/fraction of inspired oxygen ratio
